# In Pursuit of the Parietal Cell – An Evolution of Scientific Methodology and Techniques

**DOI:** 10.3389/fphys.2019.01497

**Published:** 2019-12-12

**Authors:** Vanessa Baratta, Jason Own, Chiara Di Renzo, Jenna Ollodart, John P. Geibel, Maria Barahona

**Affiliations:** ^1^Department of Surgery, Yale University School of Medicine, New Haven, CT, United States; ^2^Department of Surgery, Oncology and Gastroenterology, Hepatobiliary Surgery and Liver Transplantation, Padua University, Padua, Italy; ^3^Department of Cellular and Molecular Physiology, Yale University School of Medicine, New Haven, CT, United States

**Keywords:** parietal cell, methodology, *in vitro* techniques, gastric gland isolation, *in vivo* confocal imaging

## Abstract

The stomach has unique embryologic and anatomic properties, making the study of the parietal cell technically challenging. Numerous individuals have devoted decades of research to unraveling the pathophysiological basis of this cell type. Here, we perform a scoping review of novel *in vitro* and *in vivo* methodology pertaining to the parietal cell. First, we evaluate early *in vitro* methods of parietal cell analysis. This section focuses on three major techniques: gastric gland isolation, parietal cell isolation, and parietal cell culture. We also discuss parietal cell physiology and pathophysiology. Second, we discuss more contemporary efforts involving confocal microscopy and gastric organoids, a new technique that holds much promise in unveiling the temporal-spatial dynamics of the cell. Finally, we will discuss findings from our laboratory where we identified an active gastric vacuolar H^+^-ATPase as a putative mechanism for refractory GERD. Overall, this review aims to highlight the major milestones in understanding an elusive yet important cell. Though in no way comprehensive, we hope to provide a birds-eye view to the study of this unique cell type in the gastrointestinal tract.

## Introduction

**FIGURE 1:**
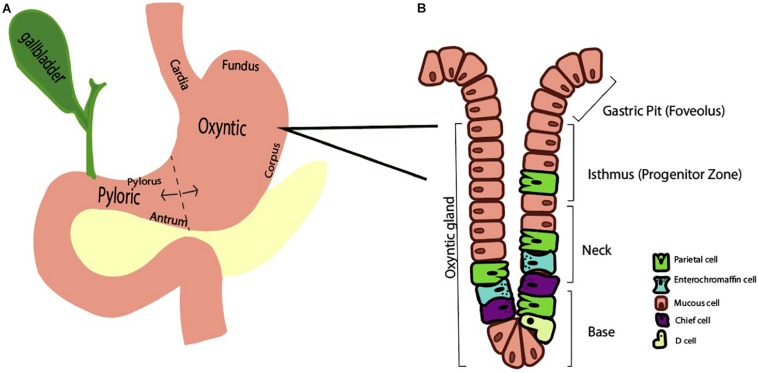
Schematic representations of the stomach **(A)** and oxyntic gland **(B)**. **(A)** The stomach is divided into two functional regions, the oxyntic region (composed of the cardia, fundus, and corpus) and the pyloric region (composed of the antrum and pylorus). **(B)** A representative oxyntic gland from the oxyntic region of the stomach features a gastric pit, isthmus, neck, and base. A variety of stomach cell types exist, including parietal cells, enterochromaffin cells, and mucous cells.

The human stomach is composed of approximately one billion parietal or oxyntic cells (Naik et al., 1971). The stomach itself is lined by columnar epithelium and punctuated by various gastric glands where specialized cells reside. These specialized cell types that are regionally distributed based on the anatomic region. Broadly, the stomach has two functional units (Schubert, 2012). The larger, oxyntic region comprises the cardia, fundus, and corpus and accounts for 80% of the stomach, whereas the pyloric region comprises the remaining 20% via the pylorus and antrum (Figure 1A). As the name implies, the oxyntic portion of the stomach is where the oxyntic or parietal cell resides. A gastric gland is divided into the pit, isthmus, neck, and base and has at least five specialized cell types based on location (Figure 1B). In the oxyntic fundus or body, parietal cells are particularly abundant and secrete acid. Neighboring cells in the gland may include chief cells, enterochromaffin cells, D cells, and mucous cells.

**FIGURE 2:**
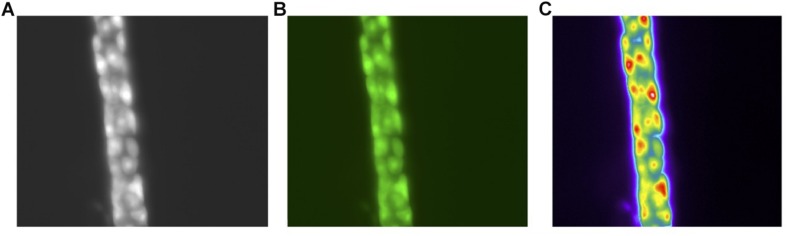
Images of the human gastric gland **(A)**. Phase contrast of the human gland at 40× Magnification **(B)**. pH sensitive dye BCECF-selective loading in parietal cells **(C)**. Pseudo color image of the parietal cells following stimulation with a secretagogue. All images acquired with a Olympus OM2 microscope.

A single parietal cell can concentrate hydrogen up to 4 million times, from pH 7.4 – 0.8 (Rabon et al., 1983). It undergoes a series of well-orchestrated events to generate HCl through the H^+^, K^+^ ATPase and the associated Cl^–^ and K^+^ channels (Schubert, 2012). When stimulated, tubulovesicles fuse with the membrane to form an extensive network of canaliculi, increasing the surface area of the parietal cell’s apical membrane (Sachs, 2003; Sachs et al., 2007). Following stimulation, the H^+^, K^+^ ATPase appears in the microvilli. When stimulation ceases, the canaliculi break down and the tubulovesicles are restored, as the parietal cell transitions back to the non-secretory state. These changes have been captured at the ultrastructural level. Figure 2 demonstrates BCECF-loading of parietal cells from our laboratory (Figures 2A–C, unpublished). Parietal cells not only govern gastric acid secretion, but also produce growth factors crucial for cell maturation. Our current knowledge of the parietal cell, however, has evolved from rudimentary beginnings.

In the early 1800’s, people were fascinated by stomach digestion but were puzzled by the agent responsible for degrading food matter (Baron, 1979). Since food could not be broken down by common acids, it was postulated that the stomach possessed a certain “vital force” (Kousoulis et al., 2012). In 1824, William Beaumont studied the gastric contents of a gunshot wound victim through a gastric fistula, further proving that the stomach generated acidic contents (Baron, 1979), Figure 3. Over a century later, little remained known about the stomach. In 1968, the editors of Gastroenterology mused, “The major questions in the field…are still unanswered. We cannot formulate the reactions within the parietal cell which result in hydrogen ion production. We are unable to state the etiology of peptic ulcer” (Davenport, 1968).

**FIGURE 3:**
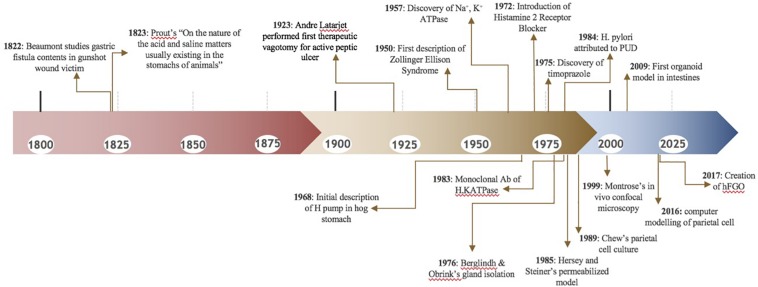
Key Events and Studies Involving the Parietal Cell and Gastric Acid Production.

Later that year, critical findings on the parietal cell began to unfold. Sachs studied hog gastric mucosa and first described the K^+^-stimulated ATPase (Sachs et al., 1976). Subsequently, a wave of studies emerged to further characterize this transporter (Ganser and Forte, 1973; Lee et al., 1974; Rabon et al., 1982). In the latter half of the twentieth century, three *in vitro* techniques emerged, permitting investigative studies on the parietal cell: (1) Gastric gland isolation. (2) Parietal cell isolation. (3) Parietal cell cultures.

As we shine a spotlight on supporting gastrointestinal cell types, we hope to illuminate the efforts that have led to our current understanding of the parietal cell. Figure 3 and Table 1 may serve as visual guides during the narrative.

**TABLE 1:** Pros and cons of parietal cell methodology.

**Technique**	**Pros**	**Cons**
Gastric gland isolation	– Able to study smallest functional unit of stomach	– Indirect method of measuring H^+^ secretion
		– Collagenase affected transport proteins
		– Also contains non–parietal cells
Parietal cell isolation	– Easy to manipulate pH, ion composition w/o diffusion barrier	– Time consuming
	– Study on a single cell type	– Inconsistent parietal cell yields
		– Chemical digestants damage the cell
		– Isolated parietal cells behaves differently
Parietal cell cultures	– Can attain high yields of parietal cells	– Loss of parietal cells over time
	– Study on a single cell type	– Unable to see cell–cell interactions
Permeabilized gastric gland models	– Allows for assessment of signal transduction in the Parietal cell	– Digitoxin: cells lose secretagogue sensitivity
		– Alpha toxin: only smaller proteins were permeable
*In vivo* confocal microscopy	– Allows for real–time pH monitoring during acid secretion	– Not accurate for pH < 2
	– Evaluates effects *in vivo*	
Gastric gland dissection	– Can monitor effects of various drugs on H^+^, K^+^ ATPase through changes in intracellular pH, Ca, Cl	– Restricted to *in vitro* analysis
	– Can selectively inactivate sodium and potassium dependent transporters by exposing it to 0–Na and 0–K solutions	
Gastric organoids	– A near physiological three–dimensional model	– Difficult to maintain high population parietal cells
	– More accurate representation of *in vivo* system	– Lack of immune cells, neurons, vascularization
	– Can sustain *H. pylori* microinjections	

## Gastric Gland Isolation

Berglindh and Obrink (1976) developed a method for isolating gastric glands from the corpus of rabbit mucosa, as delineated in methodology of their 1976 paper. Briefly, after appropriate anesthetization, they harvested the stomach. Next, they stripped off the corpus mucosa, minced the tissue, and exposed it to collagenase. To demonstrate gland viability, they measured oxygen consumption and evaluated electrolyte content. Gastric gland cells were identified through staining and electron microscopy (Berglindh and Obrink, 1976).

With this technique, scientists were able to expose isolated gastric tissue to various secretagogues to assess gastric acid production. For example, the effects of histamine, acetylcholine (ACh) and gastrin were found by measuring the degree of oxygen consumption in gastric glands. They also employed an aminopyrine (AP) uptake assay to indirectly measure HCl excretion (Sack and Spenney, 1982). The ratio of the accumulated ^14^C-AP in the intra- versus extra glandular compartments served as an index of parietal cell function, quantified via a scintillation counter. AP is a weak base with a pKa of 5.0. In an unprotonated state, the weak base freely diffuses across membranes. In a protonated state, the aminopyrine remains in a particular compartment. Impurities in the base and variations in centrifugation falsely lowered aminopyrine ratios and underestimated the parietal cell activity (Sack and Spenney, 1982). Nevertheless, the technique was an invaluable tool to quantify gastric acid secretion.

Overall, gastric gland isolation was an important scientific technique with advantages and disadvantages (Table 1). Gastric gland isolation was beneficial in that the tight junctions and polarity of the cells were unperturbed. As a result, scientists could study cell-cell interactions. However, the presence of collagenase affected the transport proteins and limited studies on signal transduction pathways. In addition, isolated glands contained non-parietal cells, preventing experimentation on individual parietal cells. These limitations paved the way for parietal cell isolation.

## Parietal Cell Isolation

The parietal cell is particularly challenging to isolate, due to its thick connective tissue. As a result, a combination of mechanical and chemical digestants was used to isolate the cells. Susceptibility of the gastric tissue to separation is species-dependent (Walder and Lunseth, 1963). For example, collagenase fully disperses all gastric cells in dogs and guinea pigs yet has poorer dissociating power in rabbits and rats. Pronase and EDTA are alternative agents for cellular separation. Early attempts to isolate parietal cells involved excessive damage to the cells (Walder and Lunseth, 1963; Blum et al., 1971; Romrell et al., 1975).

The first landmark study in parietal cell isolation was that performed by Soll (1978) on canine stomachs (Soll, 1978). His technique was unique in that he (a) completely isolated the mucosa from the submucosa and (b) introduced a calcium chelation step to facilitate disruption of cell junctions thereby minimizing exposure to chemical digestants.

In the first part of the experiment, the mucosa of the oxyntic part of the stomach was stripped from the submucosa and incubated in collagenase, EDTA, and collagenase again. The mucosal cells were then serially centrifuged and resuspended before being passed through a nylon mesh.

In the second part of the experiment, the parietal cell population was purified with a Beckman elutriator rotor. The rotor separated cells by counter-flow centrifugation based on varying sedimentation velocities. Parietal cell yields were obtained up to 85%.

Another method of parietal cell isolation was the Percoll method. In this technique, polyvinyl pyrrolidone-coated colloidal silica particles (Percoll, Pharmacia Fine Chemicals, Uppsala, Sweden), were mixed with isolated cells to a specified density (Pretlow and Pretlow, 2014). Afterward, the cells underwent 15 min of 190 G centrifugation in a swinging bucket. The surface layer consists of parietal cells, while the bottom layer consisted of non-parietal cells.

Overall, isolated cells are advantageous, because they are easy to manipulate in the absence of a connective tissue diffusion barrier. However, the *in vitro* technique has numerous limitations (Table 1). In the isolation process, the cell loses its polarity and its capacity to secrete HCl. In fact, many scientists preferred studying gastric glands as they were the smallest functional unit of the stomach. In addition, parietal cell isolation is time consuming and can result in cell clumping. The repeated centrifugation process concentrates the number of parietal cells but does not obtain a pure parietal cell population. To overcome some of these limitations, investigators focused on culturing cells.

## Parietal Cell Cultures

A working parietal cell culture was long sought after though difficult to attain (Chew, 1994). Even after serial centrifugation to enrich the cell pool, parietal cells dwindled with cell passages. Scientists initially hypothesized that parietal cells dedifferentiated over time. It later became apparent that the more rapidly dividing mucous cells out-populated the parietal cells.

Chew et al. (1989) were able to obtain a nearly 100% parietal cell yield by combining centrifugation and density gradient techniques. When they placed highly enriched parietal cells in culture lacking serum, the parietal cells persisted. The cells were also responsive to known secretagogues, thus exhibiting the parietal phenotype. For the parietal cells to survive, they were placed in a unique basement membrane extract called Matrigel. The cells required high water quality with a specific resistivity and inclusion of antibiotics and antimycotics (Pretlow and Pretlow, 2014). Issues with the parietal cell culture included inevitable loss of cell numbers, around 10% per day. In addition, the presence of the parietal cell was verified only indirectly through its response to known secretagogues and the accumulation of aminopyrine.

In the wake of parietal cell cultures, Forte and colleagues applied high-pressure freezing (HPF) techniques to capture ultrastructural changes during acid secretion (Sawaguchi et al., 2002). Cultured parietal cells were mounted on aluminum plates and frozen at high pressures to prevent crystal formation. Ice in the frozen specimen was substituted with an organic solvent, gradually warmed to 20°C, embedded in Epon, and cut into thin sections. The nucleus and cytoskeletal were better preserved with HPF than conventional chemical fixation. In addition, the tubulovesicle changes were captured by pre-exposure to histamine. The presence of the cell membrane in parietal cell cultures made it difficult for investigators to evaluate signal transduction pathways. Scientists sought to introduce protein fragments into the cytoplasm to evaluate downstream effects. To facilitate this, permeable parietal cell models emerged (Yao and Forte, 2003).

## Permeabilized Models

One of the earlier permeabilized models using the detergent digitoxin was introduced by Hersey and Steiner (1985). Digitoxin permeabilized the parietal cell by creating pores in the membrane to permit protein entry. A downside was that the cell was no longer sensitive to secretagogue stimulation. Subsequently, Thibideau introduced the alpha toxin permeabilized model (Thibodeau et al., 1994). Alpha-toxin was isolated from *Staphylococcus aureus* to create pores in the parietal cell membrane. Here, the parietal cell was still receptive to secretagogues while maintaining permeability. A downside was that the alpha-toxin generated narrow pore sizes, so only small nucleotides could pass through. Gentler detergents like Beta-escin were introduced that permitted entry of larger molecules without disrupting the parietal cell (Akagi et al., 1999). Overall, these three *in vitro* methods – gastric gland isolation, parietal cell isolation, and parietal cell cultures with permeabilization – paved the way for newer techniques to emerge.

## *In Vivo* Confocal Microscopy

The stomach’s ability to resist damage from its own highly acidic fluid has been a topic of interest. Through *in vivo* confocal imaging on an everted rat stomach, Montrose and colleagues quantified pH changes at the gel-mucous gastric surface (Chu et al., 1999). First, they used a surgical exteriorization technique. The stomach was exteriorized, opened along the greater curvature, and everted to expose the mucosa. Baseline luminal pH measurements of the gastric contents were recorded. The everted stomach was secured to the abdominal wall with sutures and the rat was placed prone on the microscope stage. The stomach tissue was maintained at physiological temperatures and continuously perfused with solutions of varying pH’s.

To measure pH changes in real time, they used a fluorescent extracellular dye, Cl-NERF in the perfusate. They excited the dye with an argon laser and calculated fluorescence intensity ratios of the emissions (Chu et al., 1999). As an internal reference, Lucifer yellow was added to the perfusate and Cl-NERF/Lucifer ratios were converted to various pH’s at the level of the gastric surface. As hypothesized, when the gastric mucosa was perfused with acidic solutions, the gastric surface pH was relatively alkaline. When the rats were injected with pentagastrin, the pH of the perfusate decreased, while the pH at the gastric surface remained alkaline. These studies confirmed that the gel-mucous stomach layer provides a self-protective mechanism from the acidic contents it secretes. With confocal microscopy, high resolution images were obtained at various depths of a tissue specimen without sectioning and processing. Overall, this technology has permitted significant advances in the understanding of stomach physiology.

## Parietal Cell Physiology

**FIGURE 4:**
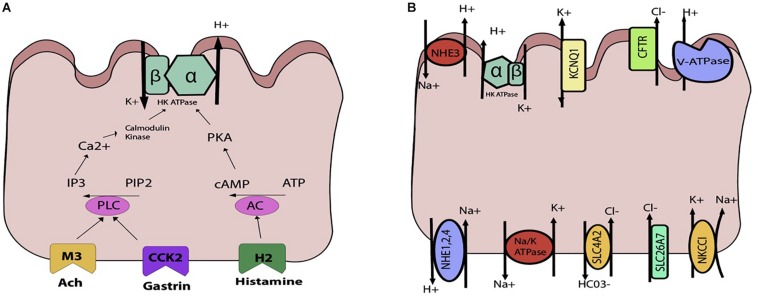
Schematic representations of the parietal cell **(A)** gastric acid stimulants **(B)** exchangers, transporters, channels. **(A)** The H, K ATPase is stimulated through three primary inputs: acetylcholine (ACh), gastrin, and histamine. ACh and gastrin bind to their receptors and trigger activation of phospholipase C (PLC), which converts PIP2 to IP3 and triggers release of Ca^2+^from the sarcoplasmic reticulum. This leads to downstream activation of the H, K ATPase. Histamine binds to the H2 receptor, triggering activation of adenylyl cyclase and activation of PKA. **(B)** Multiple apical and basolateral anion and cation movements occur to maintain the gradient and electroneutral transport of Hydrogen.

Parietal cells carry the H, K ATpase in cytoplasmic tubulovesicles that are eventually shuttled to the apical membrane through various routes of activation. Three primary pathways, the neurocrine, paracrine, and endocrine, are involved in inducing parietal cell acid secretion (Schubert, 2012). With regards to the neurocrine pathway, the neurotransmitter ACh is responsible for acid secretion. Ach directly activates the parietal cell by attaching to the muscularinic M3 receptor. This activation triggers downstream phospholipase C’s conversion of phosphatidylinositol bisphosphate (PIP2) into inositol trisphosphate (IP3), leading to release of intracellular calcium stores and activation of calmodulin kinases that activate acid secretion (Figure 4A).

In the endocrine activation pathway, antral endocrine G cells secrete gastrin which directly binds to type B cholecystokinin receptors, triggering the same pathway generating increased intracellular calcium as that of ACh. Indirectly, gastrin also stimulates histamine secretion from ECL cells.

In the paracrine pathway, enterochromaffin cell releases histamine, which separately activate acid secretion. By binding to H2-specific receptors on the parietal cell, histamine triggers a signal transduction pathway through activation of adenylyl cyclase, which increases intracellular cAMP that serves. Indirectly, histamine acts through H_3_ receptors which suppress somatostatin secretion, thereby abolishing any inhibition of acid secretion by somatostatin.

Overall, there are multiple interactions amongst ACh, gastrin, and histamine and their associated pathways to regulate acid secretion. While these are the three main mediators of acid production, a variety of other stimulants have been identified, including gastrin-releasing peptide, nitric oxide in specific amounts, pituitary adenylate cyclase activating polypeptide, ghrelin, atrial natriuretic peptide, and even coffee (Schubert, 2012).

As the parietal cell is activated by one or more pathways previously described, the hydronium ion is generated and HCl secreted. In order to maintain the H^+^, K^+^ ATPase pump, a careful interplay of channels, exchangers, and transporters is required (Figure 4B). On the apical side is the H^+^, K^+^ ATPase, which is a dimeric protein and member of the P-Type family of ATPases. The alpha subunit spans the membrane 10 times, whereas the beta subunit spans once. This transporter is typically stored in tubulovesicular elements (TVE) that fuse with the apical membrane under parietal cell stimulation to deliver the transporter machinery to the membrane (Schubert, 2012). To maintain the potassium gradient, the apical KCNQ1 channel is nearby (Heitzmann and Warth, 2007). Other apical K^+^ channels include Kir 2.1 and Kir 4.1 (Forte, 2004). The bicarbonate that is generated from the hydrogen that is extruded by the H^+^, K^+^ ATPase is generated along with H^+^ from the intracellular carbonic anhydrase reaction exits the cell through the SCL4A2. For each hydrogen that is secreted, bicarb leaves the cell basolaterally. To maintain electroneutral acid secretion, chloride is also apically secreted along with hydrogen, generating HCl. On the apical side, the CFTR allows for chloride entry into the gastric lumen (Sidani et al., 2007). Basolaterally, chloride enters the cell through SLC4A2, SLC26A7, and NKCC1 (Kosiek et al., 2007). Overall, various cations and anions are involved in transcellular movement to maintain the H^+^, K^+^ ATPase pump. Though calcium is not directly involved in transcellular movement, its release from the sarcoplasmic reticulum is essential for parietal cell stimulation. The identification of the calcium sensing receptor (CaSR) on the surface of freshly isolated rat gastric tissue has also been associated with an increase in intracellular calcium release and parietal cell activation (Dufner et al., 2005).

## Parietal Cell Pathophysiology

**FIGURE 5:**
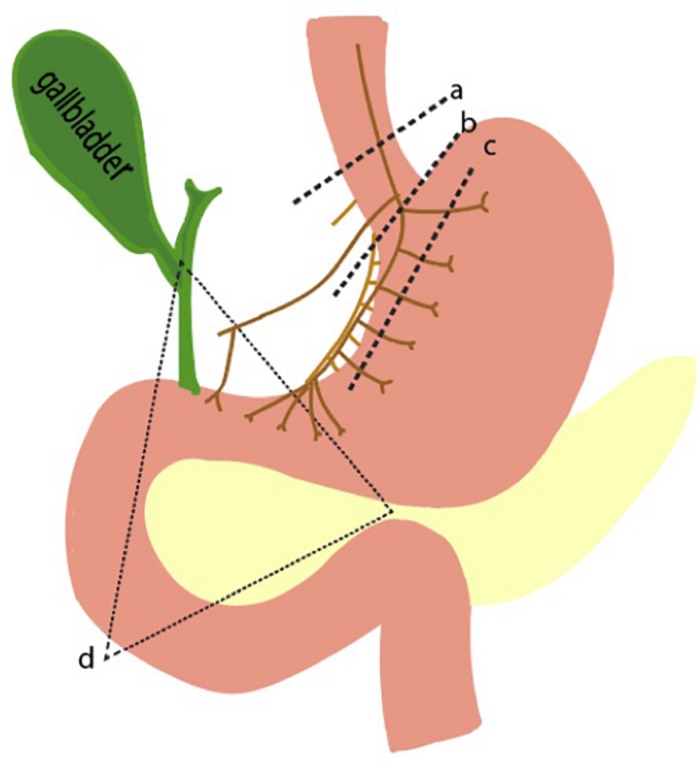
Pathophysiologic basis of gastric acid hypersecretion. Illustrated are selected examples of changes to physiological acid secretion. Lines a,b,c represent surgical transection lines for truncal, selective, and highly selective vagotomies. This reduces cholinergic input for acid secretion. Triangle d is the anatomical region where a gastrinoma is most likely to exist. A gastrinoma provides constitutively high levels of gastrin, leading to overactive parietal cells, and hyperplastic changes in morphology.

A spectrum of diseases emerges when any of the well-regulated pathways in parietal cell function go awry. There may be disorders of increased and decreased gastric acid secretion. On one end of the spectrum, gastric hypersecretion can result from various pathologic disease states, ranging from gastrinoma to peptic ulcer disease. On the other end, achlorhydria or the absence of HCl, can result from autoimmune disease states or from surgical interventions like vagotomies. As explained previously, three main inputs – gastrin, ACh, and histamine – are known to stimulate the parietal cell. Thus, perturbations of this inputs result in altered gastric acid secretion (Figure 5).

Pathophysiological elevations in gastrin can occur in patients with gastrinomas, a type of neuroendocrine tumor that can arise sporadically or as part of the familial syndrome, MEN1 (Kitagawa and Dempsey, 2005). The state of having elevated gastric acid secretion due to a gastrinoma is Zollinger Ellison syndrome, which manifests as intractable ulcers, weight loss, and abdominal pain (Isenberg et al., 1973; De Angelis et al., 2018). Surgery is the mainstay of treatment, though preoperative and intraoperative identification of the small gastrinomas can be difficult. Over 90% of tumors are found in the gastrinoma triangle, an anatomical zone defined by the junction of the pancreatic neck and body, the junction of the second to third parts of the duodenum, and the junction of the cystic and common bile duct (Figure 5).

Acetylcholine is the other main stimulator of parietal cell secretion, which has been a primary target for surgically reducing acid hypersecretion. Shown in Figure 5 are the three different types of vagotomies that can be performed: truncal vagotomy (a), selective vagotomy (b), and highly selective vagotomy (c). The more selective vagotomies leave parts of the vagus nerve, which helps facilitate gastric emptying problems associated with the truncal vagotomy. Due to the advent of proton pump inhibitors, the need for surgical vagotomies has declined.

One of the most common disease states linked to altered gastric acid homeostasis is peptic ulcer disease. Peptic ulcers are focal lesions in either the stomach or duodenum that can emerge for a variety of reasons, most commonly *Helicobacter pylori*. *H. pylori* is a spiral shaped bacterium that can protect itself from the damaging effect of nearby HCl through its local production of urease, which converts urea into ammonium and bicarbonate (Kitagawa and Dempsey, 2005). Various mechanisms by which *H. pylori* causes damage has been studied and is discussed at length in other reviews (Camilo et al., 2017; Kamboj et al., 2017. As it pertains to acid hypersecretion, *H. pylori* inhibits the antral D cells that produce somatostatin, thereby leading to hypergastrinemia and acid overproduction. Therapy for *H. pylori* include a combined course of antibiotics as well as proton pump inhibitors (PPI, Yang et al., 2014; Strand et al., 2017).

## An Explanation of Ppi Failure

A better understanding of stomach physiology has led to major advances in the treatment of gastric disease. Characterization of the H^+^, K^+^ ATPase resulted in the development of proton pump inhibitors that irreversibly inhibit the transporter. However, many patients still suffer from acid hypersecretion. Using the ammonium prepulse technique, our lab identified the vacuolar ATPase as a constitutively active H^+^-ATPase that is not susceptible to proton pump inhibitors (Kitay et al., 2018). In this experiment, we performed gastric gland dissection to isolate tissue. We then loaded parietal cells in the gastric tissue with acid and flushed them with solutions that were free of sodium and potassium in order to eliminate activity of the endogenous H^+^, K^+^ ATPase and the sodium hydrogen exchanger (NHE). We then exposed cells to omeprazole and identified persistent efflux of hydrogen, as measured by intracellular pH (pH_i_). The subsequent recovery of pH_i_ was attributable to H^+^-ATPase, the only remaining pathway for the extrusions of protons. In addition, we discovered that the activity of the H^+^-ATPase was upregulated when exposed to calcium (Kitay et al., 2018; Figure 4B).

## Gastric Organoids

Organoids are three-dimensional, stem cell-derived cell models first developed in murine intestines. In 2009, Sato placed intestinal stem cells into an extracellular matrix enriched with intestine-specific niche factors. They obtained cells that differentiated into intestinal cell types ranging from goblet to Paneth cells (Sato et al., 2009). The first gastric organoids were developed as an extension of the intestinal organoid protocol (Barker et al., 2010). Adult stem cells from murine antral glands were embedded into extracellular matrices and exposed to stomach-specific niche factors (Barker et al., 2010). Gastric organoid cells differentiated into chief, neck, pit and enteroendocrine cells. The one cell type that was elusive was the parietal cell. Though present in the initial culture, the parietal cells decreased with multiple passages.

To counteract this problem, scientists used pluripotent stem cells (PSCs). Noguchi cultured corpus organoids from mouse ESCs, which generated all three germ layers (Noguchi et al., 2015). The embryoid bodies from primordium spheroids were embedded into matrix and allowed to differentiate over 60 days with exposure to selected niche factors. These gastric organoids developed functional parietal cells. McCracken and colleagues then exposed human PSC-derived foregut progenitors to beta catenin to create human, fundic-type gastric organoids (hFGOs) (McCracken et al., 2017). From here, they exposed the hFGOs to a variety of putative parietal cell agonists and measured expression of ATP4A and ATP4B, known parietal cell markers (McCracken et al., 2017). Interestingly, mature, terminal parietal cells have dedifferentiating capacity; Notch activation in parietal cells can be de-differentiated into stem cells (Kim and Shivdasani, 2011). Other transgenic mice that have been engineered to overexpress Noggin, led to the development of dedifferentiated cells at the cost of the parietal cell lineage (Tetreault and Katz, 2012). What is most notable in transgenic studies on parietal cells is that genetic alterations more frequently lead to parietal cell loss than parietal cell gain. Overall, the embryonic properties of parietal cells remains under investigation.

Overall, organoids unite the positive aspects of both *in vitro* and *in vivo* systems in that they allow for easy experimental manipulation, while maintaining the cellular diversity of native tissue (Engevik et al., 2018). Nevertheless, it is still difficult to maintain organoids with high populations of parietal cells. New studies are evaluating the effects of *H. pylori* microinjections into gastric organoids. It is important that gastric organoids maintain parietal cells over time.

## Computer Modeling of the Parietal Cell

Another new concept in the understanding of the parietal cell is computer modeling using Berkeley Madonna Software (Crothers et al., 2016). The software is a mathematical modeling package and a differential equations solver. Crothers et al. (2016) created a dynamic model of the parietal cell based on known features of Na^+^, K^+^ATPase, AE2, and the H^+^, K^+^ ATPase. It quantitatively represents the activity of all parietal cells located within 1 cm^2^ of adult mammalian tissue. Voltage measurements from classical studies in a resting and stimulated parietal cell were entered into the software to recapitulate a model of the cell. The model adequately predicted flux and potential differences but also provided novel insight into the role of canalicular potassium in parietal cell secretion.

## Discussion

Clearly, we have made significant strides in the understanding of the parietal cell. Historically, the study of the parietal cell plagued the most ambitious and astute researcher. Since the stomach consists of a variety of cell types, it was particularly challenging to isolate the parietal cell. In this chronological review, we described some of the various methodological milestones that helped shape our current understanding of the parietal cell.

The parietal cell is a small component of the alimentary tract, yet immense research has been devoted to the topic. This phenomenon may be twofold. First, dysfunctional parietal cells lead to human disease with a large socioeconomic burden, which has galvanized research efforts (Banic et al., 2011). With the discovery of proton pump inhibitors, gastroesophageal reflux disease is now manageable. Second, the parietal cell has historically been challenging to study. The stomach has multiple cell types that are affected by autocrine, endocrine, and paracrine pathways. It is therefore difficult to dissect the underlying physiological roles. With time, scientists have built upon one another to find novel ways to study this otherwise elusive cell.

## Author Contributions

VB involved in conception, design, and writing and editing of the manuscript. JOw, CD, JOl, and MB involved in writing and editing of the manuscript. JG involved in conception, design, and editing of the manuscript.

## Conflict of Interest

The authors declare that the research was conducted in the absence of any commercial or financial relationships that could be construed as a potential conflict of interest.
